# CRISPR/Cas9 – An evolving biological tool kit for cancer biology and oncology

**DOI:** 10.1038/s41698-019-0080-7

**Published:** 2019-03-18

**Authors:** Xueli Tian, Tingxuan Gu, Satyananda Patel, Ann M. Bode, Mee-Hyun Lee, Zigang Dong

**Affiliations:** 1 Basic Medical College, Zhengzhou University, 450001 Zhengzhou, Henan China; 2 China-US (Henan) Hormel Cancer Institute, No.127, Dongming Road, Jinshui District, 450008 Zhengzhou, Henan China; 3 The Hormel Institute, University of Minnesota, Austin, 55912 USA; 4 The Collaborative Innovation Center of Henan Province for Cancer Chemoprevention, Zhengzhou, China

## Abstract

The development of genetic engineering in the 1970s marked a new frontier in genome-editing technology. Gene-editing technologies have provided a plethora of benefits to the life sciences. The clustered regularly interspaced short palindromic repeats/CRISPR associated protein 9 (CRISPR/ Cas9) system is a versatile technology that provides the ability to add or remove DNA in the genome in a sequence-specific manner. Serious efforts are underway to improve the efficiency of CRISPR/Cas9 targeting and thus reduce off-target effects. Currently, various applications of CRISPR/Cas9 are used in cancer biology and oncology to perform robust site-specific gene editing, thereby becoming more useful for biological and clinical applications. Many variants and applications of CRISPR/Cas9 are being rapidly developed. Experimental approaches that are based on CRISPR technology have created a very promising tool that is inexpensive and simple for developing effective cancer therapeutics. This review discusses diverse applications of CRISPR-based gene-editing tools in oncology and potential future cancer therapies.

## Introduction

### The CRISPR/Cas9 system

**Fig. 1:**
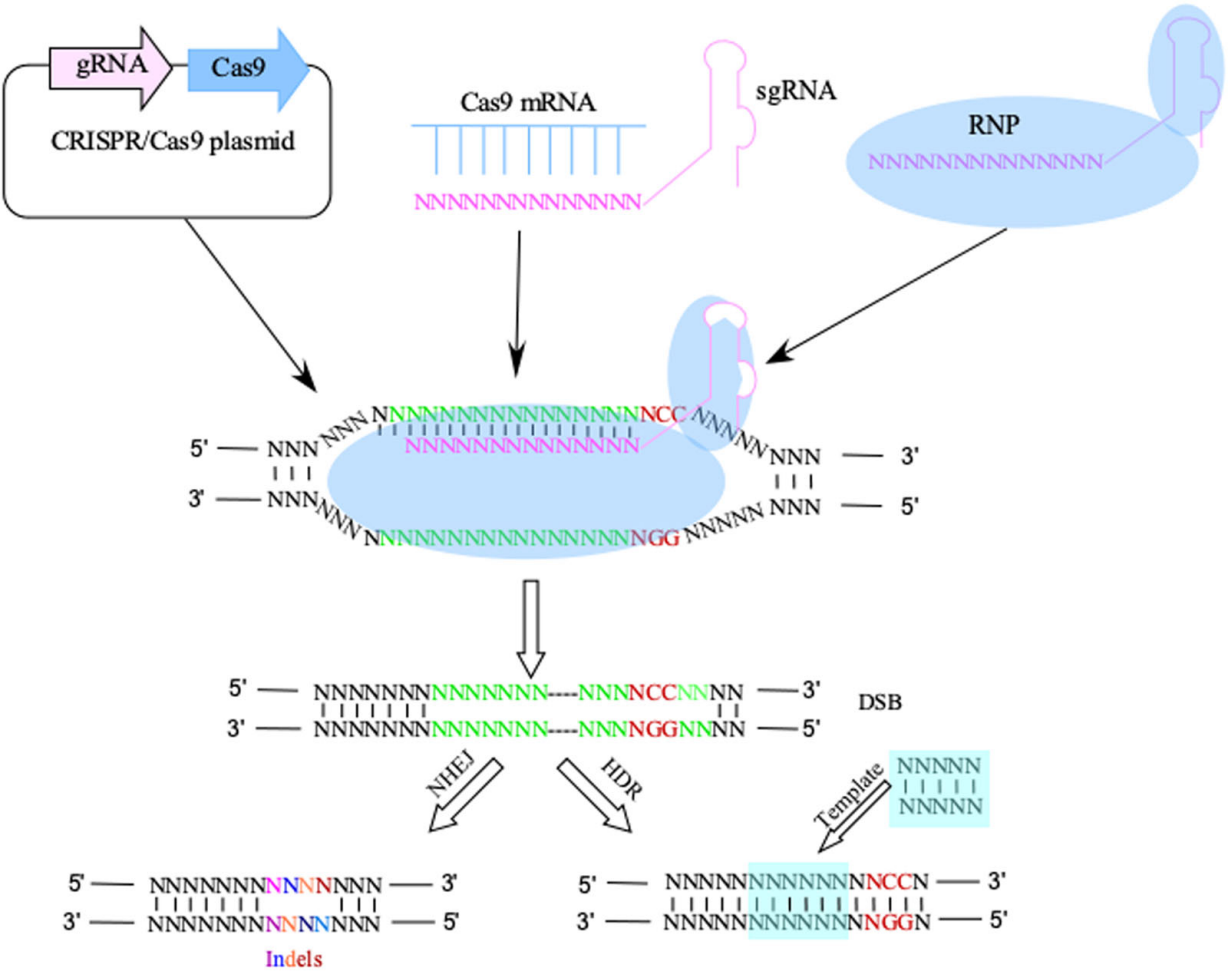
CRISPR/Cas9-based gene modification. Common methods of delivering the CRISPR system include a plasmid-based method and Cas9 protein complex with sgRNA or RNP. After the sgRNA binds to the target site of genomic DNA, the Cas9 protein creates a DSB around the PAM site. Random indels or precise modifications introduced into the genomic DNA by the NHEJ or HDR pathway

CRISPR/Cas9 is a prokaryotic, adaptive immune system that consists of a programmable RNA molecule that helps guide an associated Cas9 endonuclease to specific exogenous genetic invaders based on recognized sequences.^1^ The CRISPR-Cas9 system consists of two components, a Cas9 endonuclease and a single-stranded guide RNA (sgRNA).^2,3^ The sgRNA directs the Cas9 endonuclease to cleave both DNA strands in a sequence-specific manner (Fig. 1). DNA cleavage occurs at a sequence 3 base pairs upstream of an “NGG” protospacer adjacent motif (PAM).^4^ Following the double-strand break (DSB), the genome is repaired by DNA-DSB repair mechanisms. Using the CRISPR/Cas9 system, targeted genome modifications can be made, such as the introduction of small insertions and deletions (indels) mediated through the relatively error-prone non-homologous end-joining (NHEJ) pathway or the high fidelity homology-directed repair (HDR) pathway.^5^ Genes of interest can be easily targeted using a 17–21 nucleotide-targeting sequence. To identify genes that are important for a particular phenotype, a pooled population of sgRNAs can be introduced into Cas9-expressing cells by phenotype-based screening of genomic changes.^6^ In this review, we provide examples of current applications of this technology and speculate on future applications in cancer biology and oncology.

### CRISPR/Cas9 variations

Many variations of the CRISPR/Cas9 system have been developed (Table 1). The Cas9 protein consists of a bi-lobed architecture and the sgRNA is captured between the alpha-helical and nuclease lobes. In the nuclease lobe are two functional domains, HNH and RuvC. The RuvC domain belongs to the retroviral integrase superfamily of proteins and it cleaves the non-target DNA strand whereas the HNH domain cuts the targeted strand of the specific DNA. Normally, the HNH and RuvC domains generate a DSB.^7^ The inactivation of both domains by a mutation at H840A and D10A in the HNH and RuvC domains, respectively, results in a catalytically inactive Cas9 (dCas9). However, a single mutation of HNH or RuvC results in the generation of a single-strand break rather than a DSB. The Cas9 H840A and D10A mutants also have nickase activity wherein the RuV mutant D10A nicks the targeting strand and the HNH mutant H840A nicks the non-targeting strand. Because dCas9 is enzymatically inactive, it cannot cleave DNA. However, it retains its RNA-guided DNA binding ability, which has led to several innovative applications.^8^ dCas9, when fused to a transcriptional repressor peptide such as KRAB (Kruppel associated box), can be used to knockdown gene expression by guiding RNA. This fusion system can block the initiation of transcription and elongation and is referred as CRISPRi. The dCas9-KRAB fusion protein, when co-expressed with a target-specific sgRNA, binds the sgRNA, and the entire complex binds to the DNA strand, blocking the initiation of transcription and elongation resulting in depletion of transcripts of interest.^9^ In a similar approach, dCas9 can also be used to activate gene expression if it is fused with an activator peptide such as the VP64 and VPR activation domains. This complex is called CRISPRa and can increase transcription of target gene transcripts. CRISPRi and CRISPRa provide new tools for investigating human genome functions, transcriptome research, and regulation of functional factors in cancer biology and oncology. This differs from the canonical CRISPR system that often causes meaningless mutations or leads to a chaotic phenotype.^10^ Compared with other CRISPR approaches, dCas9-based CRISPRi and CRISPRa are inducible, reversible, have fewer off-target effects, and low toxicity. These approaches have advantages in long non-coding (lnc) RNA knockdown and overexpression.^11^ In cancer research, precise regulation of gene expression is a very useful approach and scientists have developed and expanded different systems, such as RNAi (RNA interference) and ORF (open reading frame) expression for loss or gain-of-function studies.^11^ RNAi has played a critical role in biological studies mainly because it has deterministic outcomes and is easy to deliver into mammalian cells. On the other hand, CRISPR system-based tools are often difficult to deliver into mammalian cells. Two components are required compared with a single component RNAi. However, RNAi can also lead to unpredicted non-specific toxicity and strong siRNA/shRNA can cause extensive off-target effects.^12^ Thus, even though CRISPR has disadvantages, CRISPR-based loss-of-function approaches are widely used because CRISPRi shows less endogenous off-target effects compared to RNAi, and also provides a high specificity of gene knockdown by blocking distinct promoters. It can also be useful for targeting lncRNA, whereas RNAi may be inefficient.^13^

**Table 1:** Variations of the CRISPR system

Variations	Features	Effects	Advantages	Disadvantages	Applications in cancer research
CRISPR/Cas9	WT Cas9; sgRNA	Double-strand break at the target site	Versatile; effective; stable; easy accessibility	Off-target; PAM limited; different modified alleles	Set up research model; functional gene study; drug target identification
CRISPR/Cas9 Nickase	Mutant Cas9 H840A or D10A; sgRNA	Single-strand break	Convenient; efficient; flexible; precise, scalable; robust	PAM limited; 2 sgRNA for KO	Manipulate epigenetic modifications; Simultaneous activation and repression
CRISPRi	dCas9; repressor peptide; sgRNA	Block transcription elongation or knockdown transcripts	Inducible; reversible; low off-target effects; low toxicity	PAM limited; off-target effects at bidirectional promoters	Genome and transcriptome research; LncRNA knockdown and overexpression
CRISPRa	dCas9; activator peptide; sgRNA	Increase transcription		PAM limited; complicated to deliver the multiple components	

### CRISPR/Cas9 and basic research

**Fig. 2:**
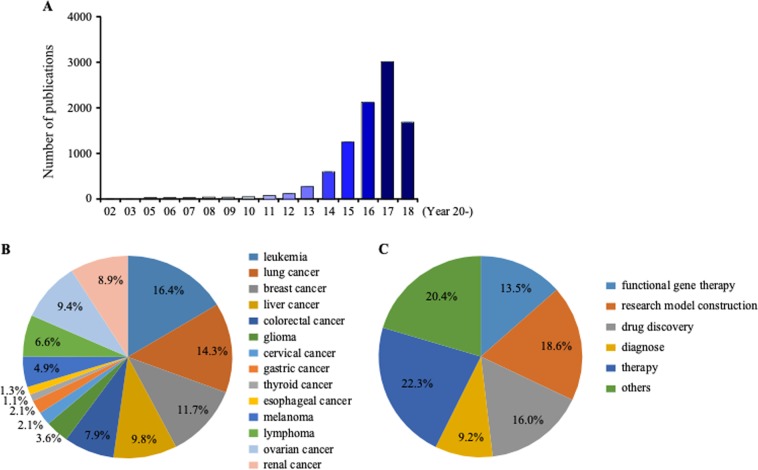
Publications focusing on CRISPR identified from 2002 to 2017 on PubMed reflect great interest and cancer applications. **a** The number of publications on PubMed with the keyword “CRISPR” from 2002 to 2018. **b** CRISPR/Cas9 applications in different cancers as reported on PubMed. **c** CRISPR/Cas9 applications with different approaches for cancer. Both **b** and **c** results are shown as a percentage

CRISPR/Cas9 is a rapidly developing gene-editing tool that has revolutionized many areas of research. An online search ranging from 2002 until 2018 (5/26/2018) was conducted on PubMed using the term “CRISPR/Cas9” (Fig. 2). The search results revealed an increasing number of research articles (9332 publications) on PubMed over those years (Fig. 2a). In 2017, publications regarding CRISPR/Cas9 numbered 2889. Among these publications, 8.6% are associated with cancer and most of those focus on leukemia (16.4%), lung cancer (14.3%), breast cancer (11.7%), or liver cancer (9.8%, Fig. 2b). A search was also conducted using key words “CRISPR/Cas9” and “cancer” along with “model”, “functional gene”, “drug target”, “diagnosis”, or “therapy”. Most papers were focused on cancer therapy (22.3%), model construction (18.6%), drug validation (16%), or functional gene studies (13.5%; Fig. 2c). Clearly, since its initial discovery, the CRISPR/Cas9 system has been integrated into cancer research as a useful tool to identify oncogenes and mediators of cancer.^14^

The occurrence and development of cancer is a highly complex process with multi-gene and multi-path interactions.^15^ Many scientists have been attempting to decipher the mechanisms of cancer occurrence, development, and metastasis and clearly, CRISPR has accelerated research efforts.^16^ Presently, CRISPR technology is used to investigate the genetic mechanisms in almost all areas of cancer, from prevention to prognosis and treatment,^14,17^ which greatly promotes transition to the clinic. For example, CRISPR has been applied to breast cancer diagnosis, treatment, and even drug resistance research.^18^ Based on the CRISPR-dCas9 system, researchers have fused a DNA methyltransferase effector to dCas9 and infected it by lentivirus into healthy breast cells.^19^ The results showed that the *cyclin dependent kinase inhibitor 2A (CDKN2A)* gene is a key driver in causing abnormally rapid cell division, which might be an early diagnostic marker in breast cancer. Another group attempted to apply the CRISPR system in breast cancer therapy by targeting the *HER2* gene.^20^ Results showed that targeting *HER2*-expressing cells inhibited growth and attenuated tumorigenicity, compared to non-targeted cells, suggesting a new therapeutic choice against breast cancer. Additionally, CRISPR was also used to confirm the BRCA1-delta11q alternative splice isoform as a primary factor in breast cancer resistance to treatment.^21^ The CRISPR/Cas9 system is becoming a widespread, practical, and useful tool against many types of tumors and it promises to accelerate cancer research.^14^

## Applications of CRISPR/Cas9 in cancer research

### Generation of cancer models

Cancers are driven by processes influenced by underlying genes.^22^ Being able to decipher the molecular genetics of disease is crucial to elucidate the underlying mechanisms.^23^ Cell lines and animal models are invaluable for dissecting the relationship of the genotype, chemotherapeutic effects, and immune microenvironment. CRISPR genetically engineered cancer models now can be produced rapidly, efficiently, and inexpensively^24^ (Table 2). Leukemia models were generated by targeting several inactivated genes through a lentivirus-delivered Cas9–sgRNA system in primary hematopoietic stem and progenitor cells (HSPCs).^25^ The pooled lentiviruses target several genes, including *Tet2, Runx1, Dnmt3a, Nf1, Ezh2*, and *Smc3*. With a fluorescent marker, multiple targeted HSPCs were selected that are involved in the development of myeloid malignancy. The CRISPR/Cas9 technology has been used to generate several other cancer models.^26^ By introducing mutations of *APC*, *SMAD4*, *TP53*, *KRAS*, and/or *PIK3CA*, an organoid model of colon cancer was built with CRISPR technology.^27^ In the future, the generation of precision cancer models would greatly stimulate the study of functional cancer genomics and enhance the development of precision cancer medicine.^28^

**Table 2:** CRISPR applications in cancer research

Application	Targets	sgRNA design	Vehicle for delivery	Features	Advantages	Disadvantages
Generate cancer model	HSPCs; healthy human organoids	Targeting the model type-related suppressors oncogenes	Pooled lentivirus	Disrupt suppressors or edit oncogenes	Rapid, efficient, and inexpensive	Special delivery techniques; tissue limited
Synergistic gene study	Cells	Targeting optional drug target from database	Lenti-double sgRNA library	Together with deep sequencing	Effective, low cost, innovative approach	Double sgRNA construction; need highly efficient sgRNA; special analysis
Target validation	Drug or anticancer reagent resistant cells	Lentiviral library from Addgene; or optional targets	Plasmid	Identify the target from resistant cells by sequencing	Effective	False-positives
Gene diagnose	Genome	Target sensitive genes	Lentivirus	Together with Cas13a or Cas12a to induce collateral effects	Sensitive, rapid, low cost	Certain template concentration

### Synergistic gene studies using CRISPR/Cas9

The CRISPR/Cas9 system also provides an effective strategy for the identification of synergistic gene interactions, which could be used to block drug resistance. A CRISPR-based double knockout (CDKO) system in K562 leukemia cells has been developed using a double sgRNA library system to screen for combinatorial genes and identify pairs of synthetic lethal drug targets. Together with deep sequencing, phenotype measurement and gene analysis have identified interactions between synergistic drug targets, like *BCL2L1* and *MCL1*.^29^ Another simple and efficient strategy called CombiGEM (combinatorial genetics en masse)-CRISPR was also developed to analyze combinatorial gene function.^30^ It is similar to the CDKO system in which two pooled sgRNA libraries were combined in one vector. With this approach, some genetic hits (e.g., *KDM6B* + *BRD4)* were discovered. Depleting these genes using the CombiGEM system has shown stronger synergistic efficacy against ovarian cancer cell proliferation compared to a reported small-molecule inhibitor.^30^ Compared to drug inhibition, the CRISPR system costs less.^9^ The power of the CRISPR library to screen functional variants could thus play an important role in precision cancer medicine.^31^ Indeed, this innovative approach could allow for the development of personalized genotype-based therapies built upon genotype-specific targets.^32^

### Functional gene screening using CRISPR/Cas9

Precision cancer medicine has resulted in the development of many targeted drugs to treat different cancers. Targeted therapy already has shown enormous potential but several challenges still exist. Only patients who exhibit a certain mutation or altered gene expression respond to the targeted drug treatment and drug resistance to the therapy still occurs.^33^ Functional genome-screening approaches using the CRISPR system could reveal gene expression changes after treatment and pinpoint genes associated with resistance to the targeted drugs, thereby identifying new biomarkers for precision therapy and providing new insights into cancer development.^34^ One successful example involved screening for a cancer metastasis-related gene with a CRISPR-Cas9-mediated loss-of-function screen.^35^ This group infected a non-metastatic lung cancer cell line with a mouse genome-scale CRISPR knockout (mGeCKO) sgRNA library and the transduced cells were subcutaneously transplanted into immunocompromised mice. Six weeks later, the mice exhibiting lung cancer metastasis were selected for sequencing the enriched sgRNA. Finally, several candidate genes associated with lung metastasis were identified and validated, including the already reported genes *Pten*,^36^ *miR-152*,^37^ and *miR-345*,^38^ and several new genes like *Nf2, Trim72, and Fga*. Other loss-of-function screens were also applied to examine suppressor genes in liver tumors.^39^ In this study, p53^−/−^/Myc mouse embryonic liver progenitor cells were infected with an mGeCKO library and transplanted into nude mice. sgRNAs that were increased 8-fold were chosen as candidates and *Nf1*, *Plxnb1*, *Flrt2*, and *B9d1* were identified as new tumor suppressors involved in liver cancer formation. Another group applied CRISPR interference (CRISPRi) to screen for functional lncRNA loci, which could modify cell growth.^40^ They designed a comprehensive sgRNA library to target the lncRNA transcription start site (TSS). The library was transduced into several different cell lines and together with sequence analysis, 499 lncRNAs were identified as associated with cell growth.

CRISPRi can repress the transcription of targeted genes by recruiting the complex of dCas9 and a repressor to the TSS, which is more suitable for lncRNA gene research.^10^ In summary, combining CRISPR-based functional genetic screening is a powerful approach to validate alternative genes associated with a specific phenotype.^41^

### Target validation by CRISPR/Cas9

Revealing the mechanism of action for small-molecule drugs is a time-consuming and laborious process.^42^ Efficiently identifying new drug targets with a CRISPR-Cas-based genetic screening system (CRISPRres) containing large sgRNA libraries is now possible.^43^ If the molecular binding site is depleted or mutated, cancer cells commonly acquire resistance, but the molecular target could be clearly identified by sgRNA sequencing. CRISPR was used to successfully identify nicotinamide phosphoribosyl transferase as the primary target of KPT-9274, an anticancer agent. Based on CRISPR/Cas9 technology, another team set up a system called DrugTargetseqR to identify direct physiologic targets by mutating potential targets for drug resistance.^44^ In this study, kinesin-5 was confirmed as the direct target of ispinesib by mutating kinesin-5 D130V or A133P in HeLa cells. Targeting the exons that encode functional protein domains rather than the 5′ exons reportedly is a better method using the CRISPR system for identifying drug targets.^45^ In this case, a negative selection system was constructed with a GFP reporter and used to screen hundreds of chromatin regulatory domains in leukemia cells. The sgRNAs targeting of functional domains, like the ATPase domain or DNA-binding domain, led to a stronger negative selection phenotype compared to targeting the 5′ exon. Obviously, combining CRISPR genome-editing with deep sequencing or cellular biophysical assays could produce new insights into target validation studies.^46,47^

### Gene diagnosis

Genetic diagnostics to determine sensitive genes is critical for cancer prevention.^48^ Although a low frequency mutation is not easily determined by sequencing, a CRISPR-based diagnostic system called SHERLOCK (Specific High Sensitivity Enzymatic Reporter UnLOCKing), has been established.^49^ A key factor in this system is Cas13a, an RNA-guided RNase, which induces robust non-specific single-stranded DNA (ssDNA) trans-cleavage as a collateral effect.^50^ Another essential element is the reporter signal, which is released after RNA cleavage. This method has been used to detect two cancer mutants, BRAF V600E and EGFR L858R, and appears to be a highly sensitive detection approach. A similar system referred to DETECTR (DNA endonuclease-targeted CRISPR trans reporter) has been developed.^51^ In this system, another Cas family member, Cas12a, is used, and acts similar to Cas13a. An additional enzyme, RPA (recombinase polymerase amplification), is used to amplify micro-samples. RPA can be used as a detection tool for screening for infections in cancers. The system was used to detect HPV types 16 and 18 in lung carcinomas and appears to be a rapid and inexpensive approach.^52^

## Anticancer applications in clinical trials

Based on promising results of pre-clinical studies, the CRISPR/Cas9 system could also potentially be used clinically to target cancer-causing genes. At this time, eleven clinical trials are underway testing the effectiveness of CRISPR for cancer therapies (Table 3). Seven of the eleven trials are immunotherapies that target program cell death-1 (PD-1) protein expression. The PD-1 protein and programmed cell death ligands (PD-Ls) are important for the negative regulation of the immune system, specifically on T-cells. Their attenuation of the immune response helps tumor cells survive by evading the immune system.^53^ Pembrolizumab, a monoclonal antibody against PD-1, confirmed that blocking PD-1 and PD-L1 in the immune system could significantly increase the overall survival rate in cancer patients.^54^ PD-1 is thus an attractive target for immunotherapy and PD-1 inhibitors have been recently approved by the U.S. Food and Drug Administration (FDA) for cancer immunotherapy. Coincidentally, a team in China has gone one step further by using CRISPR/Cas9 to directly target PD-1 in patients (NCT02793856). Using CRISPR/Cas9, they disabled PD-1 expression in cells harvested from a metastatic non-small-cell lung cancer patient. They expanded the cells in a large culture system and then injected the modified cells back into the patient.^55^ Based on the results of a dose-escalation study, the safety of PD-1 knockout-engineered T-cells in treating metastatic non-small cell lung cancer will be evaluated. Similar trials targeting PD-1 expression in T-cells are being conducted in prostate (NCT02867345), bladder (NCT02863913), and renal cell cancers (NCT02867332). Another phase II clinical study has applied the same PD-1 knockout on T-cells for esophageal cancer (NCT03081715).

**Table 3:** Anticancer applications in clinical trials

Applications	Target site	Study phase	Editing strategy	Clinical trials identification
Advanced esophageal cancer	PD-1	Phase II	PD-1 knockout	NCT03081715
Castration resistant prostate cancer	PD-1	Phase I	PD-1 knockout	NCT02867345
Muscle-invasive bladder cancer	PD-1	Phase I	PD-1 knockout	NCT02863913
Metastatic non-small cell lung cancer	PD-1	Phase I	PD-1 knockout	NCT02793856
EBV associated malignancies	PD-1	Phase I Phase II	PD-1 knockout	NCT03044743
Metastatic renal cell carcinoma	PD-1	Phase I	PD-1 knockout	NCT02867332
Relapsed or refractory leukemia and lymphoma	CD19 and CD20 or CD22	Phase I Phase II	Edit CD19 and CD20 or CD22	NCT03398967
Human papillomavirus-related malignant neoplasm	HPV16-E6/E7 HPV18 E6/E7	Phase I	HPV16-E6/E7 or HPV18 E6/E7 knockout	NCT03057912
CD19 + leukemia and lymphoma	TCR B2M	Phase I Phase II	TCR and B2M knockout	NCT03166878
Tumor of the central nervous system	NF1	—	Fix NF1 mutation allele	NCT03332030
Multiple myeloma Melanoma Synovial sarcoma Myxoid/round cell liposarcoma	TCR PD-1	Phase I	TCR and PD-1 knockout	NCT03399448

Generating chimeric antigen receptor (CAR) T-cells by CRISPR/Cas9 is another ex vivo approach in clinical trials. Researchers from the University of Pennsylvania organized the first-in-human trial to test the effect of HLA-A*0201 restricted NY-ESO-1 redirected engineered T-cells in a wide range of cancer types, including relapsed refractory multiple myeloma (MM), melanoma, synovial sarcoma, and myxoid/round cell liposarcoma (NCT03399448). Tumor rejection activity might be enhanced by eliminating endogenous TCR and PD-1 with the use of CRISPR. Another clinical trial (NCT03166878) focused on CD19+ leukemia and lymphoma. Allogeneic CD19-directed CAR T-cells were generated using the lentiviral delivery of the CAR receptor and CRISPR RNA by electroporation to disrupt the endogenous *TCR* and *B2M* genes. This approach might assist in evading host-mediated immunity and thus deliver anti-leukemic effects to patients without having to worry about graft-versus-host-disease (GVHD). Unfortunately, a subset of patients relapse due to the loss of CD19 in tumor cells. Thus another clinical trial is focusing on CRISPR-edited dual specificity CD19 and CD20 or CD22 CAR T-cells, which could recognize and kill the CD19-negative malignant cells through recognition of CD20 or CD22 (NCT03398967). This may be a complementary approach for a wide range of patients. In another application, CRISPR/Cas9 was used to disrupt the human papillomavirus-16 (HPV16) E7 protein, an oncogenic protein that is important for the maintenance of the malignant phenotype in cervical cancer. When E7 was disrupted in HPV-positive cervical cancer cells, inhibition of tumor growth inhibition, and induction of apoptosis occurred.^56^ These promising results have led to a phase I clinical trial (NCT03057912) to evaluate the safety and efficacy of TALEN-HPV E6/E7 and CRISPR/Cas9-HPV E6/E7 in treating HPV persistent and HPV-related cervical intraepithelial neoplasia. In this study, a CRISPR/Cas9 plasmid targeting HPV16-E6/E7T1 or HPV18 E6/E7T2 was administered twice a week for four weeks to disrupt the expression.

Another clinical trial using CRISPR technology has been designed to screen and identify drugs (NCT03332030). In this trial, an induced pluripotent stem cell (iPSC) bank was established from phenotypically well-characterized patients with Neurofibromatosis type 1 (NF1). NF1 is a frequent neurocutaneous syndrome which easily causes various benign or malignant tumors.^57^ To identify a NF1 specific target drug, CRISPR/Cas9 was used to develop different cell lines, *NF1* wild type (*NF1*^+/+^), *NF1* heterozygous (*NF1*^+/−^), and *NF1* homozygous (*NF1*^−/−^). By examining the reverse or alleviated phenotypes, *NF1*-specific drugs might be identified. Although results from CRISPR/Cas9 clinical studies might be promising, more work is needed to assure that CRISPR/Cas9 is a safe and effective tool for treating human cancers.

## Future applications

Cancer is characterized by numerous genetic alterations and multiple mutations. The CRISPR system provides unparalleled precise control to correct cancer-associated mutations in the genome compared with the original ZFNs or TALENs technologies.^58,59^ One could easily imagine that directly correcting abnormal genes through the HDR pathway will be an effective therapeutic strategy against cancer.^60^ Recent work has provided strong evidence for this vision. *TMEM135-CCDC67* and *MAN2A1-FER* fusion genes have been identified as cancer-derived genes in human prostate cancer and hepatocellular carcinoma.^61,62^ These genes were replaced by the *HSV1-TK* death-promoting gene using CRISPR-Cas9 technology. HSV1-TK is a phosphotransferase that can block DNA synthesis as a suicide gene.^63^ In this study, an adenoviruses delivery system was used to disrupt the dysfunctional gene by dCas9 and two sgRNAs. One sgRNA was designed to target the gene locus and the other was targeted to the breakpoint region of the fusion gene. In this way, CRISPR-Cas9 only induces DSBs in cancer cells (not normal cells) that contain the *TMEM135-CCDC67* and *MAN2A1-FER* fusion genes, which greatly reduced the off-target effects. The DSBs were repaired by an *HSV1-TK* suicide gene template, which contained the same flanking sequence as the HDR donor.^63^ With this method, the *TMEM135-CCDC67* and *MAN2A1-FER* fusion genes will be inactivated or replaced by the *HSV1-TK* suicide gene with no side effects on normal cells.

Another promising application of the CRISPR/Cas9 system is personalized therapy, which requires rapid and systematic screening to identify the genotype-specific changes in the patient’s genome.^64^ Combining guide RNA libraries with Cas9 nuclease could assist in screening a broad range of potential gene targets and identify genes responsible for the altered phenotypes, including genetic mutations and dysfunctional signaling pathways, which might initiate cancer or promote progression.^65^ Therapeutic strategies can subsequently be developed based on the results of the gene screening. The treatment of EGFR-mutant lung cancer is a typical example for personalized therapy.^66^ One method is to target the tumor-driving mutations by tyrosine kinase inhibitors (TKIs). Because of the inevitable drug resistance problems, other “molecular surgeries” using CRISPR technology to directly correct or disrupt the mutated site to inactivate the oncogenic activity, might be a more permanent strategy for EGFR-mutant lung cancer treatment.^67^ The CRISPR technology is poised to open the door to effective personalized cancer treatment from specific applications of genome screening to therapeutic strategies.^68^

The CRISPR-Cas9 system is a new rapidly developing technology with potential to completely revolutionize scientific research including transformation from basic research to actual clinical application providing more opportunities for a greater understanding of cancer biology and treatment.

## Conclusions and perspectives

The advent of CRISPR technology has revolutionized the biological sciences and provided cancer biologists with a powerful gene-editing method that can alter the genetic make-up of cells in unprecedented ways. Indeed, promising results have been achieved in diverse disciplines, from basic research to the development of potential therapies against cancer, congenital defects, and other chronic diseases. Many challenges associated with CRISPR technology still exist, mainly in clinical use associated with delivery and safety.^69^ Improved strategies will be required to increase the targeting efficiency and to minimize off-target effects. Ensuring that CRISPR/Cas9 has precise genome-editing ability is also important. Following CRISPR/Cas9 mediated DSBs, DNA repair can be achieved by either the “error-prone NHEJ” or “precise repair through HDR” in the genome. In an effort to promote HDR over NHEJ, a study by Maruyama et al. used the NHEJ inhibitor Scr7 and observed that Scr7 treatment did indeed increase the levels of HDR-mediated genome editing over NHEJ.^70^ In addition to concerns regarding off-target effects, considerations regarding the immune response to CRISPR-mediated gene-editing must also be considered. The Cas9 protein is bacterial in origin and thus might elicit an immune response, which could in turn affect its gene-editing efficiency. Also, the exogenous sgRNAs may be cleared by immune cells like monocytes and macrophages.^71^ The delivery approach for CRISPR/Cas9 thus depends upon the objective as well as the target. Transient expression of the sgRNA and the Cas9 protein through microinjection might be safer than other non-viral or viral delivery methods. Viral delivery has the highest efficiency for the expression of the Cas9 protein and sgRNA, but comes with risks. During one in vivo study, the lentivirus particles elicited a strong immune response in treated animals, which affected the efficacy of the lentiviral vector.^72^ Another issue with lentiviral delivery is also the possibility that the lentiviral vector could randomly integrate into the cellular genome. To overcome this issue, integrase-deficient lentiviral vectors, nanoparticle carriers, or an inducible system could be used for delivery of CRISPR/Cas9.

The prohibitively high costs for CAR T-cell therapy have made this treatment unavailable to a large section of society. CAR T-cell therapy offered by Novartis, the first approved by the FDA, costs $475,000 per treatment; however, further advances in CRISPR technology might help reduce costs and make CAR T-cell therapy available to more patients. So far, the majority of CRISPR clinical trials are being conducted in China. A recent article in the Wall Street Journal suggested that this may be due to the fact that fewer rules and restrictions exist in China. Indeed, Dr. Shixiu Wu from the Hangzhou Cancer Hospital, who was featured in the article,^73^ has been able to use CRISPR technology-based treatments on his cancer patients, without the need for national regulatory approval and has few reporting requirements. Many scientists worry that the technology has the potential to harm patients and that unintended consequences from using CRISPR on patients without sufficient oversight could hinder progress in the whole field. Dr. Wu recognized that he was undertaking risks in using CRISPR-based treatments for his cancer patients, but was considering their limited available survival time. The thought is that being able to live for additional time is better than imminent death. Persistent post-treatment monitoring of patients could help to eliminate or treat any unwanted consequences. Indeed, treatment-related observations could potentially save many lives of those who are on the brink of death.

We need consider the limitation as seriously as the potential benefits of this technology. A new human trial, in which the team took a crash course in bioethics and created CRISPR babies, brought the ethical issues and controversies into the public. One hundred and twenty-two Chinese scientists and many other scientists worldwide have already condemned the trial. How to use this powerful without overstepping the ethical bottom line is a serious question that needs careful consideration. We might expect more positive reports from clinical trials as well as the development of improved approaches that will bring new hope for personalized therapy.
